# Retraction: In vivo NCL targeting affects breast cancer aggressiveness through miRNA regulation

**DOI:** 10.1084/jem.2012095006112026r

**Published:** 2026-06-30

**Authors:** Flavia Pichiorri, Dario Palmieri, Luciana De Luca, Jessica Consiglio, Jia You, Alberto Rocci, Tiffany Talabere, Claudia Piovan, Alessandro Lagana, Luciano Cascione, Jingwen Guan, Pierluigi Gasparini, Veronica Balatti, Gerard Nuovo, Vincenzo Coppola, Craig C. Hofmeister, Guido Marcucci, John C. Byrd, Stefano Volinia, Charles L. Shapiro, Michael A. Freitas, Carlo M. Croce

Vol. 210, No. 5 | https://doi.org/10.1084/jem.20120950 | April 22, 2013

This article has been retracted at the request of the *JEM* editors. The article was published on April 22, 2013, and then Fig. 1, E and F, was corrected on January 19, 2017. *JEM* was notified by the Ohio State University in January 2021 that an investigation by the College of Medicine Investigation Committee (COMIC) determined that the RNU6 data in the original Fig. 1, E and F, as well as in the corrected versions, were falsified. An Expression of Concern was issued on October 3, 2022. After further review, the editors no longer have confidence in the data and are retracting the article. *JEM* notified all authors for whom contact information could be found but received no response.
